# Pain of Chronic Sacro-Iliac Joint Atrhopathy: Managed Successfully With Conventional Bipolar Radiofrequency Procedure: A Case Report

**DOI:** 10.5812/kowsar.22287523.3583

**Published:** 2012-01-01

**Authors:** Awisul Ghazali, Gautam Das, Khaled Horani, GS Anand Kumar, Palak Mehta, Debjyoti Dutta

**Affiliations:** 1 Institution Daradia, the Pain Clinic, Kolkata, India

**Keywords:** Pain, SacroIliac Joint, Anaesthetics

## Abstract

**Background:**

Chronic sacroiliac (SI) joint pain constitutes 16% to 30% of the total prevalence of chronic low back pain, which is commonly unilateral. Apart from conservative management, various interventional pain management procedures have been reported. Intraarticular deposteroid injection has been described as the most evidence-based, but different various radio frequency (RF) procedures have been described with varied success. Conventional bipolar RF is relatively new in the management of SI joint pain. We have successfully managed pain of the SI joint origin.

**Case Report:**

A 53-year-old female who presented with unilateral back pain with radiation to the leg was diagnosed with pain from SI joint arthropathy by clinical and diagnostic interventional procedures. She was treated conservatively without any result. Deposteriod gave good but very short-term relief. She underwent a bipolar RF procedure. An RF needle was placed at the L5 medial branch, and 2 were placed on each lateral side of the sacral foramina for the lateral branches of the S1, S2, and S3 nerve roots. Conventional RF was performed at 80°C for 90 seconds.

**Discussion:**

This case report supports the use of bipolar RF nerve ablation for chronic sacroiliac joint pain that does abate with deposteroid injection. In this patient, the Rt L5 medial branch nerve was ablated using conventional RF technique, followed by conventional bipolar RF nerve ablation for the S1, S2 and S3 lateral branches. We recommend the use of bipolar RF nerve ablation for chronic sacroiliac joint pain that has an inadequate response to deposteroid injection.

## 1. Introduction

Chronic sacroiliac joint pain contributes to 16% to 30% of the total prevalence of chronic low back pain (1, 2). Most patients will complain of low back pain on one side, which is worsened by prolonged sitting. The pain is primarily nociceptive in nature.

Reports in the literature consider multiple intraarticular deposteroid injection as having the best level of evidence in treating pain of sacroiliac joint arthropathy [Level 1B+ = one randomized controlled trial (RCT) or more RCTs with methodological weakness, demonstrate effectiveness; the benefit clearly outweighs the risk and burdens] (1).

Other interventional techniques that are becoming popular in treating resistant sacroiliac joint pain are conventional radio frequency (RF) and pulse RF of the nerves that innervate the sacroiliac joint. Both techniques are no better than multiple deposteroid injections.

A relatively new technique of ablating the sensory nerves innervating the sacroiliac joint is bipolar RF ablation (2). Since there is no clear evidence on the bipolar ablation, we report a case of successful bipolar RF nerve ablation in managing chronic sacroiliac joint pain in a 53-year-old female who had inadequate responses to serial deposteroid injections. 

## 2. Case Report

RMA, a 53-year-old female, was diagnosed with right chronic sacroiliac joint pain after she attended pain clinic to get further treatment. Her history revealed a chronic dull and throbbing pain that was a typical nociceptive type of pain in her right lower back that was worsened by prolonged sitting, persisting for nearly 2 years. Standing and walking temporarily reduced her pain, but the low back pain returned if she did either too long. Initially, the pain reduced with paracetamol, but later, she needed more potent non-steroidal anti-inflammatory drugs (NSAIDs); the initial attending doctor had her switch to tramadol to avoid the side effects of NSAIDs, as she needed to take the medication for long durations.

When first examined at pain clinic, the pain score was moderate to severe, with pain score ranging from 5-7/10 [using visual analog score (VAS)], nociceptive in character. On examination, there was no sensorineural deficit in the lower limbs, and during the flexion abduction and external rotation (FABER) test of the right leg, significant pain was noted in the buttock area. The shearing test was also positive in the right sacroiliac joint area. Relevant examinations to exclude other possible diagnoses, such as lumbosacral x-ray, and blood tests were performed, and the results were within the normal range.

With a high-index clinical diagnosis of chronic sacroiliac joint arthropathy, the patient was advised to receive a diagnostic joint block with local anesthetic. After giving written consent, the patient was prepared for a right sacroiliac joint block by fluoroscopy. Four milliliters of 1% preservative-free lignocaine was injected, and the patient claimed that her pain decreased by more than 50% after 5 minutes. Appreciating the 50% pain reduction as a positive result, another 40 mg of depomedrol was injected, and the patient was discharged content. However, the pain relief was not long lasting, and the patient returned to clinic after 4 weeks with a similar complaint of right lower back pain. A second injection of 40 mg depomedrol with 4 mL of 1% lignocaine was given, with good results.

After another pain-free episode of about 4 weeks, the patient experienced a similar attack of pain and came to clinic requesting another type of pain treatment. Bipolar RF nerve ablation was offered to her, and she agreed to proceed with it. The bipolar RF technique of her right sacroiliac joint was carried out on July 25, 2011. The procedure involved ablation of the right L5 medial branch and the S1, S2, and S3 lateral branch nerves. The procedure was successful, and at the follow-up at 2 weeks, 6 weeks, and 12 weeks, the patient claimed to have a pain-free back at 12 weeks. 

### 2.1. Method of Bipolar Radio Frequency Nerve Ablation Technique in Sacroiliac Joint Pain

The patient lies prone with a pillow under the abdomen during the procedure. Strict aseptic technique is applied. A C-arm fluoroscopic machine is adjusted accordingly in the antero-posterior (AP) view to obtain the best images of the right ala of the sacrum (for the L5 medial branch nerve) and the S1, S2, and S3 neural foramen. A local anesthetic was then injected at the area perpendicular to the right ala of the sacrum and just lateral to the S1, S2, and S3 foramen (3-5).

Using a 10-cm RF needle with a 10-mm active tip, the right L5 medial branch nerve was initially targeted. The final location of the needle tip was at the junction between the superior articular process of the sacral bone and the ala of the sacrum. The other target points were the lateral branch nerves of S1, S2, and S3. The tips of the needles were placed about 0.5 cm lateral to the S1, S2, and S3 foramens for each nerve (2).

Sensory stimulation at 50 Hz, 0.5 V is then tested, and the patient should feel paresthesia at a painful area. Needle tips should be adjusted accordingly if there is no paresthesia felt by the patient. Next, motor stimulation at 2.0 V and 2 Hz is carried out. There should not be any muscle contraction except for the lumbar multifidus muscle on stimulation of the L5 medial branch (3).

**Figure 1.**
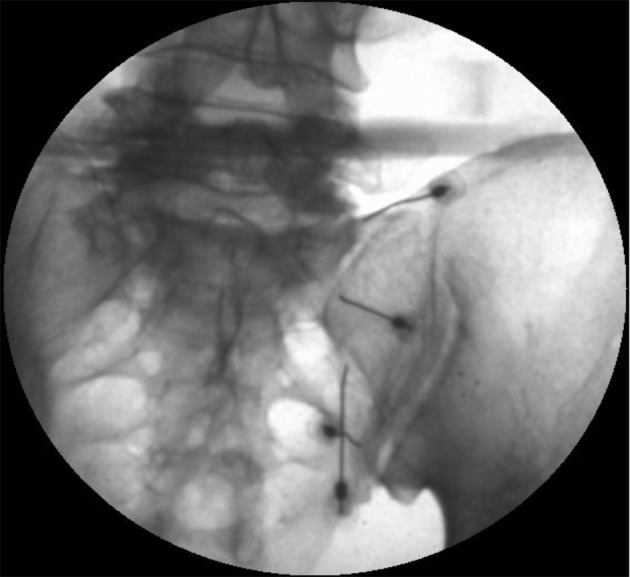
RF Needles Inserted at ala of the Sacrum and Lateral to S1, S2 Foramen, Other Needles Were Placed Similarly.

Conventional RF for 90 seconds at 80°C is carried out for the L5 medial branch nerve. For the S1, S2, and S3 lateral branch nerves, an extra RF needle is injected around 0.5 cm above each needle in a sequential manner for the bipolar technique. Using the bipolar mode for each level, 2 RF needles are stimulated concomitantly each time. A RF wave is delivered for 90 second at 80 °C for each level. Figure 1 shows needles being injected at L5 medial branch nerve and lateral branch of S1 and S2 nerves, another needle is targeted at 0.5 cm above to S1 lateral branch for bipolar technique.

## 3. Conclusions

The diagnosis of chronic sacroiliac joint arthropathy is made primarily by history and clinical examination. Multiple manual provocation tests are the clinical examinations that are used to guide the diagnosis, and the presence of 3 positive ‘manual provocation tests’ has more than 80% specificity (1). Such investigations as x-ray, magnetic resonance imaging (MRI), and blood investigations are performed mainly to exclude other diseases, such as ankylosing spondylitis and infiltrating tumor. Until now, fluoroscopy guided intraarticular local anesthetic injection is the most reliable method of establishing a diagnosis. Pain reduction of more than 50% after injection is considered positive (4).

The most common painful sacroiliac joint that presents in the clinic is a mechanical lesion associated with trauma, as well as adjacent lumbar athropathy and fusion (3). Local anesthetic, mixed with a depo-steroid injection, has the highest level of evidence in treating sacroiliac joint pain (Level 1B+ = one RCT or more with methodologic weakness, demonstrates effectiveness. The benefit clearly outweigh risk and burdens) (1).

However, in certain conditions, even though a local anesthetic mixed with a depo-steroid injection gives significant pain relief, the duration of action is short. In this case report, both injections gave significant pain relief, but the duration was around 4 weeks. For this particular reason, other techniques have been developed to reduce sacroiliac joint pain.

RF technique is the most popular technique of reducing the occurrence of pain in sacroiliac joint arthropathy. However, due to the anatomical complexity of the sacroiliac joint as well as the nerves supplied, multiple techniques of RF ablation have been tried. Generally, the sacroiliac joint can be divided into 2 parts, the dorsal side and the ventral side. The ventral side is innervated by L5 to S2, and the dorsal side is innervated by S1 to S3. 

Buijs and colleagues described techniques of ablating the dorsal root ganglion for the S1, S2, and S3 foramen and ablating the medial branch nerve for the L5 spinal nerve using conventional RF (4). Another technique is pulse RF for the same target. Both techniques carry a level of evidence of 2C+ (Effectiveness only demonstrated in observational studies only, given no conclusion). 

The bipolar method of RF ablation is a relatively new technique for sacroiliac joint pain. This technique is not targeting the dorsal root ganglion of S1, S2, and S3 but targets the lateral branches instead. Whereas a monopolar configuration drives RF current between an electrode’s exposed tip and a distant ground pad, a bipolar configuration drives RF current between 2 nearby electrode tips (2). Due to the 2-needle technique for bipolar RF, the area of nerve tissues being ablated is theoretically wider than with other techniques.

This case report supports the use of bipolar RF nerve ablation for chronic sacroiliac joint pain that does not show prolonged pain reduction to depo-steroid injections. In this patient, the Rt L5 medial branch nerve was ablated using a conventional RF technique, followed by conventional bipolar RF nerve ablation for the S1, S2, and S3 lateral branches. We recommend the use of bipolar RF nerve ablation for chronic sacroiliac joint pain that shows an inadequate response to depo-steroid injections.
